# ﻿An unusual species of *Pseudophanias* Raffray from West Tianmu Mountain, China (Coleoptera, Staphylinidae, Pselaphinae)

**DOI:** 10.3897/zookeys.1129.95245

**Published:** 2022-11-10

**Authors:** Zi-Wei Yin, Tie-Xiong Zhao

**Affiliations:** 1 Laboratory of Systematic Entomology, College of Life Sciences, Shanghai Normal University, Shanghai 200234, China Shanghai Normal University Shanghai China; 2 44 East Genta Road, Zhuji, Shaoxing, Zhejiang 311800, China Unaffiliated Zhuji China

**Keywords:** East China, new species, *Pseudophanias*, taxonomy, Zhejiang

## Abstract

The genus *Pseudophanias* Raffray includes 19 species distributed in the Oriental Region. Here, the first species from continental China, *P.tianmuensis* Yin & Zhao **sp. nov.**, is described and illustrated from West Tianmu Mountain. This species can be readily separated from all congeners by the coarse vestiture, along with its male features. A previous key to *Pseudophanias* species of East and South Asia is modified to accommodate the new species.

## ﻿Introduction

With greatly reduced maxillary palpi and often a C-shaped aedeagus, the genus *Pseudophanias* Raffray is an easily recognizable group among the Oriental genera of the ant-loving beetle tribe Tmesiphorini (Staphylinidae: Pselaphinae). After removing two species from former *Chandleriella* Hlaváč (now a junior synonym of *Pseudophanias*; Hlaváč 2000; Yin 2019), the genus currently comprises 19 species distributed in the Oriental Region (Inoue et al. 2020; Inoue and Nomura 2020, 2021). Members are usually taken from the forest leaf-litter layer, in decomposing wood, and sometimes also with ants or termites. One species from Nepal was collected in a cave and possesses an exceptionally elongate body and appendages that are typical for a cavernicolous beetle (Yin et al. 2015).

In 2006 an unidentified pselaphine collected by our friend Xiao-Bin Song from a decomposing log on West Tianmu Mountain was made available for study. At first look, the beetle hardly recalled a *Pseudophanias* because of its unusual coarsely punctate body surface, subquadrate elytra, and cylindrical antennal clubs which are unknown among all previously described members of the genus. In the following 12 years, two more specimens were collected in the same area; one was sifted from leaf litter, and the other was, again, found in dead wood with termites. This species is confirmed to belong to *Pseudophanias* and is formally described here.

## ﻿Materials and methods

The type material of the new species is deposited in the Insect Collection of Shanghai Normal University (**SNUC**), Shanghai, China. The label data of the material are quoted verbatim.

Dissected parts were mounted in Euparal on plastic slides pinned with the specimen. The habitus image of the beetle was photographed using a Canon 5D Mark III camera with Canon MP-E 65 mm f/2.8 1–5× macro lens, with a Canon MT-24EX Macro Twin Lite Flash as the light source. Images of the morphological details were produced using a Canon G9 camera mounted to an Olympus CX31 microscope under reflected or transmitted light. Zerene Stacker (v. 1.04) was used for image stacking. All images were modified and grouped into plates using Adobe Photoshop CC 2020.

Measurements were taken as follows: total body length was measured from the anterior margin of the clypeus to the apex of the abdomen; head length was measured from the anterior margin of the clypeus to the head base, excluding the occipital constriction; head width was measured across the eyes; the length of the pronotum was measured along the midline, the width of the pronotum equals pronotum’s maximum width; the length of the elytra was measured along the suture; the width of the elytra was measured as the maximum width across both elytra; the length of the abdomen is the length of the dorsally exposed part of the abdomen along its midline, the width is abdomen’s maximum width. Since one male paratype is completely disarticulated, only the holotype and the female paratype were measured. Abdominal tergites and sternites are numbered following Chandler (2001), in Arabic (starting from the first visible segment) and Roman (reflecting true morphological position) numerals, e.g., tergite 1 (IV), or sternite 1 (III). Paired appendages in the description of the new species are treated as singular.

## ﻿Taxonomy

### ﻿ Pseudophanias tianmuensis

Taxon classificationAnimaliaColeopteraStaphylinidae

Yin & Zhao sp. nov.

9C307C40-F6D7-554F-BBAF-D9AFC8979F96

https://zoobank.org/4476E74C-644F-4667-8AF5-45C75D21638A

**Figure 1.**
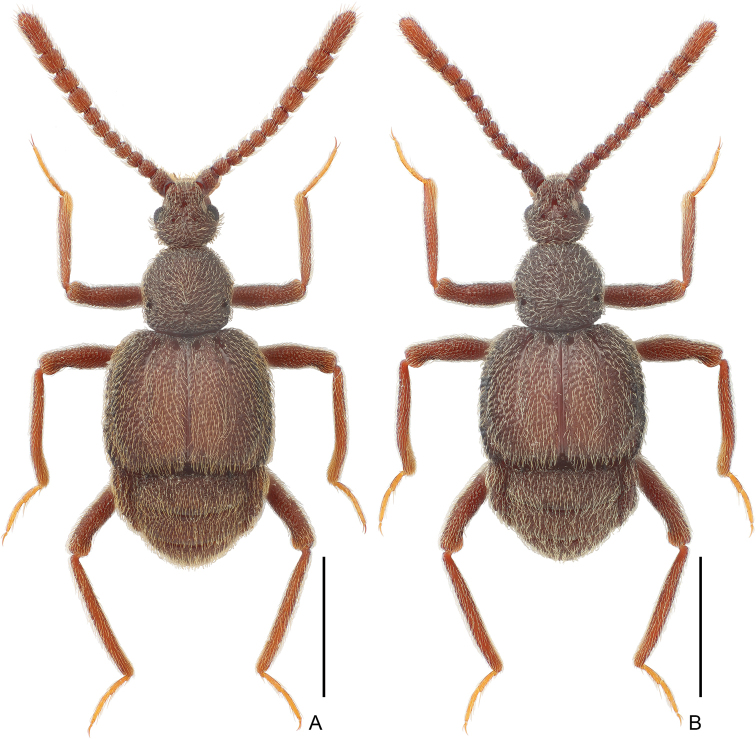
Habitus of *Pseudophaniastianmuensis* sp. nov. **A** male **B** female. Scale bars: 1.0 mm.

**Figure 2.**
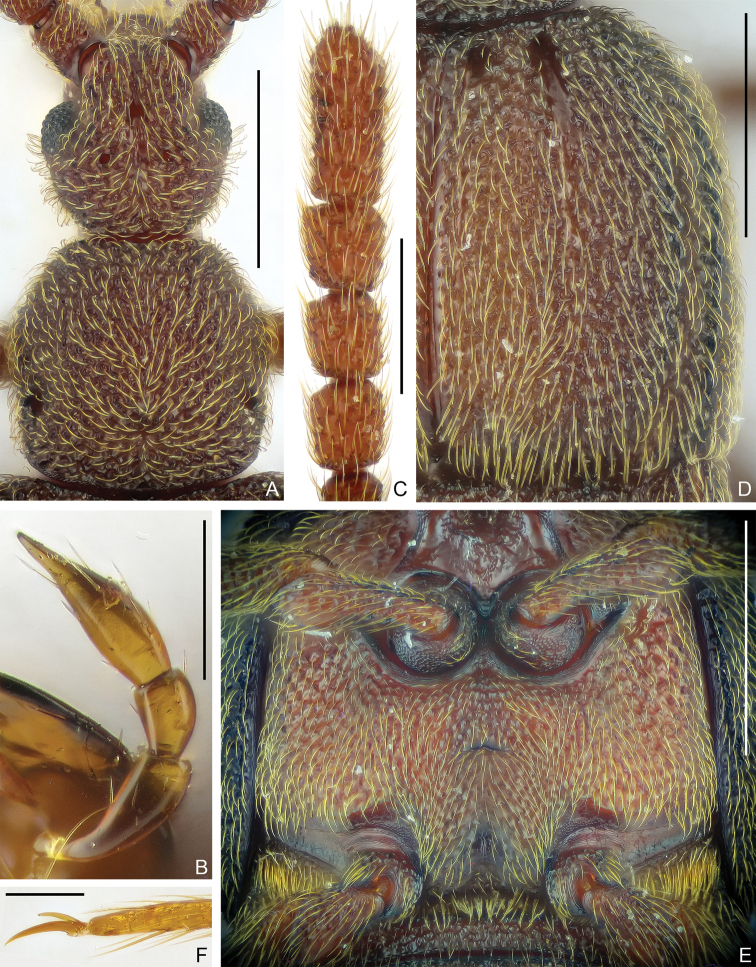
Morphology of *Pseudophaniastianmuensis* sp. nov., male **A** head and pronotum **B** maxillary palpus **C** antennal club **D** elytron **E** meso- and metaventrite **F** Mesotarsal claws. Scale bars: 0.5 mm (**A, D, E**); 0.1 mm (**B, F**); 0.3 mm (**C**).

**Figure 3.**
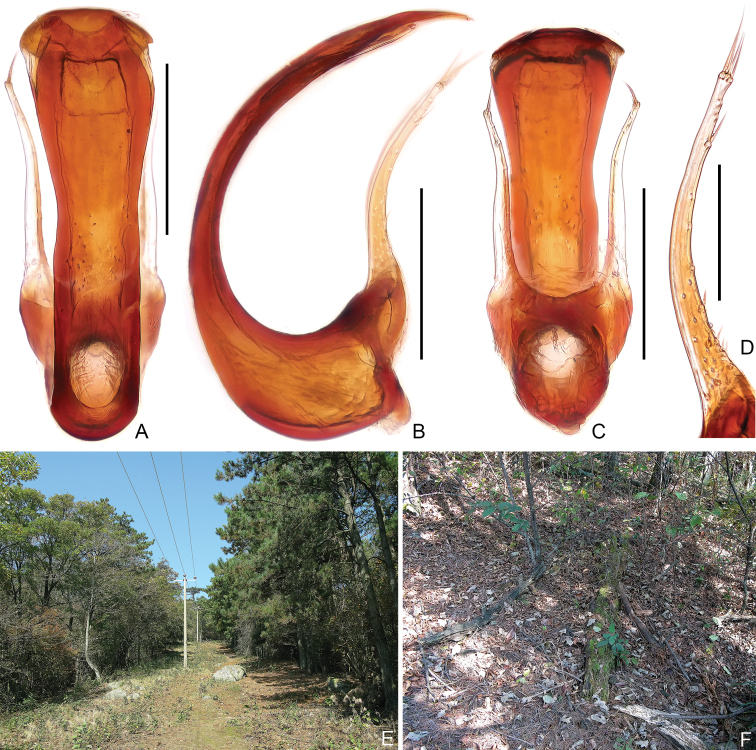
Morphology and habitat of *Pseudophaniastianmuensis* sp. nov. **A–C** aedeagus **A** dorsal **B** lateral **C** ventral **D** paramere, lateral **E** general environment of the collecting site **F** decomposing tree branches inhabited by the beetle. Scale bars: 0.2 mm (**A–C**); 0.1 mm (**D**).

Figs 1 , 2 , 3 Chinese common name: 天目隐须蚁甲

#### Type material.

(3 exx.). ***Holotype*: China**: ♂, ‘China: Zhejiang, Linan District, West Tianmu Mountain, 1100 m, 23.viii.2014, *Cryptomeria* leaf, sift, Tie-Xiong Zhao leg., 浙江省临安市西天目山(此路不通)’ (in SNUC). ***Paratypes*: China**: 1 ♂ [completely disarticulated specimen], China: Zhejiang, Linan District, West Tianmu Mountain, 1150 m, 2.vii.2006, decomposing log, sift, Xiao-Bin Song leg., 浙江省临安市西天目山(停车场); 1 ♀, ‘China: Zhejiang, Linan District, West Tianmu Mountain, 1180 m, 18.viii.2019, decomposing log, sift, Zi-Wei Yin leg., 浙江省临安市西天目山(消防道)’ (both in SNUC).

#### Diagnosis.

Body length 2.8–2.9 mm; dorsal vestiture coarsely punctate and densely setose. Head subrounded at base, with small vertexal and frontal foveae; antennomeres moniliform. Elytra with discal and lateral longitudinal ridges, areas between ridges impressed. Tergite 1 (IV) longer than 2 (V) at middle but clearly shorter than 2 and 3 (VI) combined. **Male**: antennal clubs formed by apical four enlarged, cylindrical antennomeres. Aedeagus symmetric dorso-ventrally; median lobe C-shaped laterally; parameres thin and elongate, each with two long apical and one preapical seta.

#### Description.

**Male.** Body (Fig. 1A) length 2.91 mm; color red-brown, tarsi and mouthparts lighter. Dorsal vestiture coarsely punctate and covered with short, dense setae.

Head (Fig. 2A) subrounded at base, longer than wide, length 0.63 mm, width 0.49 mm; vertex weakly convex, with two small, asetose vertexal foveae (dorsal tentorial pits); antennal tubercles indistinct; frons with single small fovea, protruding anteriorly to form rostrum; clypeus sharply descending, its surface coarse, anterior margin rounded and slightly carinate; lacking ocular-mandibular carina. Venter with two small, well-separated gular foveae (posterior tentorial pits), lacking median carina/sulcus. Maxillary palpus (Fig. 2B) small in size, palpomere 1 minute, 2 pedunculate basally and broadened apically, 3 roundly trapezoidal, 4 broadly at approximately basal 1/3 and gradually narrowing apically. Compound eyes moderately prominent, reniform in lateral view, each composed of approximately 55 ommatidia. Antenna elongate, length 1.75 mm, distinct club (Fig. 2C) formed by greatly enlarged antennomeres 8–11 and approximately as long as antennomeres 1–7 combined (0.88 mm *vs* 0.87 mm); antennomere 1 large, subcylindrical, 2–7 moniliform, of similar shape, 8, 9 and 10 each much larger than 7, subquadrate, 11 longest, slightly narrower than 10 and as long as 9 and 10 combined.

Pronotum (Fig. 2A) approximately as long as wide, length 0.66 mm, width 0.63 mm, widest anterior middle; sides from widest point greatly convergent anteriorly and subparallel posteriorly; disc distinctly convex to form hump at middle, lacking carina or sulcus; with small asetose median and lateral antebasal foveae; lacking basolateral foveae. Prosternum with anterior part much shorter than coxal part at middle, with small, asetose lateral procoxal foveae; hypomeron lacking ridge that demarcating it from pronotum; margin of coxal cavity non-carinate.

Elytra much wider than long, length 0.96 mm, width 1.24 mm, truncate at bases; each elytron (Fig. 2D) with two moderately large, asetose basal foveae; with one discal and one lateral ridge, discal stria formed by broad impressed area between ridges; humeral region lacking subhumeral fovea nor marginal stria; epipleural area broad; posterolateral margin with broad notch. Metathoracic wings fully developed.

Mesoventrite (Fig. 2E) with rough surface, with pair of thick admesal carinae, lateral area fully demarcated from metaventrite, openings of median mesoventral foveae widely separated, foveae greatly extended internally, lateral mesoventral foveae unforked, extended internally and overlapped with median foveae; intercoxal process short. Metaventrite (Fig. 2E) coarsely punctate, greatly prominent medially and with small crest; with large, asetose lateral mesocoxal foveae; posterior margin roundly emarginate at middle.

Legs elongate; femora coarsely punctate; each tarsus (Fig. 2F) with one major and one reduced pretarsal claw.

Abdomen widest at lateral margins of tergite 1 (IV), length 0.86 mm, width 1.15 mm. Tergite 1 (IV) slightly less than twice as long as 2 (V), with broad, deep basal sulcus and two basolateral foveae at lateral ends of sulcus, 2 and 3 (VI) successively shorter, lacking basal sulcus nor fovea, 4 (VII), much longer than 3, narrowed posteriorly, with one pair of basolateral foveae; tergites 1–3 each with pair of quadrangular and 4 with pair of triangular paratergites, 5 (VIII) broadly rounded, with one pair of small basolateral foveae, posterior margin roundly emarginate at middle. Sternite 2 (IV) longest, with densely setose basal sulcus and pair of basolateral foveae at lateral ends of sulcus, 3 (V) and 4 (VI) each short at middle, combined approximately as long as 5 (VII), 3–5 lacking sulcus nor fovea at base, 6 (VIII) transverse, posterior margin slightly protruding at middle, 7 (IX) composed of pair of membranous lamellae.

Aedeagus (Fig. 3A–C) 0.51 mm long, dorso-ventrally symmetric; median lobe C-shaped in lateral view and broadened in apical half in dorso-ventral view, with large basal capsule and oval foramen; parameres (Fig. 3D) each thin, elongate and curved ventrally in lateral view, with two apical and one preapical macroseta and four short setae near base.

**Female.** Similar to male in external morphology (Fig. 1B). Antenna slightly shorter, club less enlarged (0.77 mm for antennomeres 1–7 vs. 0.82 mm for antennomeres 8–11); each compound eye composed of approximately 43 ommatidia; elytra slightly shorter; metathoracic wings also well developed; metaventrite lacking crest at middle. Measurements (as for male): body length 2.84 mm; length/width of head 0.62/0.49 mm, pronotum 0.63/0.65 mm, elytra 0.91/1.22 mm; abdomen 0.83/1.13 mm; length of antenna 1.59 mm.

#### Comparative notes.

This species is unique among all known members of the genus in the coarsely punctate body surface, and a rather different type of the male antennal modifications.

#### Bionomics.

The male holotype was collected by sifting *Cryptomeria* (Cupressaceae) leaves, while the other two individuals were found inside decomposing wood with termites. Although indicated by a set of morphological traits such as the compact body segments and moniliform antennomeres, it is still inconclusive as to whether the beetle has an obligate association with social insects, pending further observations in the field or at the laboratory.

#### Distribution.

East China: Zhejiang.

#### Etymology.

The specific epithet refers to West Tianmu Mountain, the type locality of this species.

#### Remarks.

To accommodate the new species, the key to East and South Asian species of *Pseudophanias* by Inoue et al. (2020) is modified as follow:

**Table:** 

0	Whole body surface roughly punctate and with dense, short setae; antennal club formed by enlarged, subcylindrical antennomeres 8–11; elytra roundly quadrate, broadly truncate at bases	***Pseudophaniastianmuensis* Yin & Zhao, sp. nov. (East China: Zhejiang)**
–	Body surface usually finely punctate (head with coarse punctation in *P.excavatus* Inoue, Nomura & Yin) and with moderately long pubescence; antennal club formed by antennomere 11 alone, or by variously modified antennomeres 5–11, 8–11, or 9–11; elytra roundly triangular, and more strongly constricted at bases	**1**

## Supplementary Material

XML Treatment for Pseudophanias tianmuensis

## References

[B1] Chandler DS (2001) Biology, morphology, and systematics of the ant-like litter beetles of Australia (Coleoptera: Staphylinidae: Pselaphinae). Memoirs on Entomology International 15: [i–x,] 1–560.

[B2] Hlaváč P (2000) *Chandleriella*, new genus of Tmesiphorini from Borneo (Coleoptera: Staphylinidae: Pselaphinae). Entomological Problems 31(1): 91–93.

[B3] Inoue S Nomura S (2020) A new species of the genus *Pseudophanias* Raffray, 1890 from Thailand (Coleoptera: Staphylinidae: Pselaphinae). Japanese Journal of Systematic Entomology 26(2): 336–339.

[B4] Inoue S Nomura S (2021) Two new species of *Pseudophanias* Raffray from Myanmar (Coleoptera: Staphylinidae: Pselaphinae). Zootaxa 4996(3): 591–599. 10.11646/zootaxa.4996.3.11 34810510

[B5] Inoue S Nomura S Yin Z-W (2020) Three new species of *Pseudophanias* Raffray from Japan and Taiwan Island, and synonymy of *Chandleriella* Hlaváč with *Pseudophanias* (Coleoptera, Staphylinidae, Pselaphinae). ZooKeys 987: 135–156. 10.3897/zookeys.987.53648 33223888 PMC7666096

[B6] Yin Z-W (2019) First record of the genus *Chandleriella* Hlaváč (Coleoptera: Staphylinidae: Pselaphinae) from China, with description of a second species. Zootaxa 4571(3): 432–438. 10.11646/zootaxa.4571.3.11 31715811

[B7] Yin Z-W Coulon G Bekchiev R (2015) A new species of *Pseudophanias* Raffray from a cave in central Nepal (Coleoptera: Staphylinidae: Pselaphinae). Zootaxa 4048(3): 446–450. 10.11646/zootaxa.4048.3.10 26624761

